# Influence of Genetic, Dietary, and Environmental Factors on Natural Killer (NK) Cell Biology and Function: Interplay Between NK Cell Activity and Cancer Onset or Progression

**DOI:** 10.3390/cancers17182946

**Published:** 2025-09-09

**Authors:** Kawaljit Kaur

**Affiliations:** ImmuneLink, LLC., Riverside, CA 92508, USA; drkawalmann@g.ucla.edu; Tel.: +1-5093395967

**Keywords:** NK cells, preneoplasia, cancer, obesity, gene knockout, osteoclasts

## Abstract

The connection between NK cells and cancer offers valuable insights into disease management. Suppressing NK cells can encourage cancer growth, while cancer itself can weaken NK cell function. This review examines how genetic and environmental factors such as diet affect NK cell numbers and function during the early stages of cancer. It also explores the reduced NK cell activity in cancer-bearing mouse models and human patients. The mechanisms behind cytotoxic actions and cytokine release are analyzed across four NK cell maturation stages. This understanding highlights the potential of using healthy donor NK cells for immunotherapy, alongside the role of memory NK cells in treatments. While NK cell-based therapies show promise in studies, challenges remain in sustaining their effectiveness and durability. This review also discusses strategies to improve production and boost the efficiency of donor NK cell therapies.

## Simple Summary

The interaction between NK cells and cancer offers valuable insights for cancer treatment. NK cell suppression can promote cancer progression, while cancer can reduce NK cell functionality. This review explores how genetic and environmental factors such as diet influence NK cell levels and activity during the preneoplastic phase. It examines NK cell functions in mouse cancer models and human patients, focusing on their cytotoxic mechanisms and cytokine release throughout the four maturation stages. The potential of allogeneic healthy NK cells in cancer immunotherapy is highlighted, along with the therapeutic possibilities of memory NK cells. Although many NK cell-based therapies show promise in preclinical and clinical studies, challenges remain in sustaining their effectiveness and persistence. The review also discusses strategies to improve production processes and enhance the efficacy of allogeneic NK cell therapies.

## 1. Introduction: Natural Killer Cells

Natural killer (NK) cells, vital players in innate immunity, can target tumor and virus-infected cells without prior sensitization [1,2]. Derived from CD34+ hematopoietic stem cells in the bone marrow, NK cells are present in the spleen, liver, placenta, and peripheral blood [3]. They inhibit cancer through direct cytotoxicity, antibody-dependent cellular cytotoxicity (ADCC), and immune modulation via inflammatory cytokines and chemokines [4,5,6]. NK cells play a crucial role in immunology and aging research, protecting against malignancies, infections, and the effects of cellular aging. They destroy tumors, virus-infected, and senescent cells by releasing perforin and granzyme B, inducing necrotic or apoptotic cell death [1,7,8,9,10,11]. Cystatins and cathepsins regulate this process, with cathepsins C, H, L, and X as essential lysosomal proteases in NK cells [12,13,14]. Cathepsin C activates pro-granzymes such as granzyme B by removing inhibitory dipeptides, while cathepsin H acts as an alternative activator, and cathepsin L processes perforin for granzyme entry into target cells [15]. These enzymes prepare cytotoxic effectors within granules. Cystatins, especially cystatin F, inhibit cathepsins and reduce cytotoxicity [16]. Cystatin F, unique to NK cell lysosomes, transitions from an inactive dimer to an active monomer that inhibits cathepsins C, H, and L, limiting granzyme activation and perforin processing [16]. Elevated cystatin F levels are linked to reduced mature cathepsins, granzyme B activity, and NK cell cytotoxicity. Tumor microenvironments and immune stimuli influence cystatin F expression and activation, shaping NK cell responses [16,17,18]. Human NK cells rely heavily on FasLigand (CD178)-mediated cytotoxicity, especially against adherent cancer cells, either independently or alongside granule-mediated killing [19]. FasL plays a crucial role in the death-mediated cytotoxic pathway, inducing apoptosis in target cells as an alternative or complement to the granule exocytosis pathway (perforin/granzymes) [19,20]. FasL binds to the Fas receptor (CD95) on target cells, initiating extrinsic apoptotic signaling, which leads to cell death [21,22]. NK cell FasL expression increases with activation, such as through IL-2 or CD16 ligation [23,24].

NK cells offer a promising alternative to T cells in cancer therapy. As part of the innate immune system, they efficiently target MHC class I-deficient or -mutated cells [25]. While tumors can develop resistance, strategies such as activation, expansion, and genetic modification of NK cells enhance their antitumor capabilities and address drug resistance [26,27]. NK cells are activated through receptor–ligand interactions on target cells, independent of antigen processing [28]. In hematopoietic stem-cell transplantation (HSCT), NK cells can be infused alone or with others after enrichment. Allogeneic NK cells with inhibitory receptors, such as KIR, can eliminate cancer cells lacking proper MHC ligands [29,30,31,32,33]. Clinical trials have shown promising outcomes, including complete remission and improved survival rates, especially in acute myeloid leukemia (AML) cases [34,35]. NK cell therapies work well with treatments such as CAR T-cell therapy, and their lack of graft vs. host disease (GVHD) makes them a safe option for adoptive therapies [36,37,38,39,40]. These advancements bring new hope in the fight against cancer.

Natural killer (NK) cells play a key role in immune defense, as their dysfunction allows malignant cells to escape detection and promote tumor growth. This review highlights how NK cell activity is often compromised in both early and advanced cancer stages. Regular assessment of NK cell function is important for cancer patients, those at risk, and even healthy individuals, serving as a vital marker of immune health. The maturation and characteristics of NK cells have been studied in healthy, preneoplastic, and cancerous conditions, showing how unhealthy habits or genetic factors can impact their performance. It also examines the challenges and advancements in NK cell-based immunotherapies and explores ways to improve the success of allogeneic NK cell treatments.

## 2. Changes in the Number and Function of NK Cells at the Preneoplastic Stage or Due to Genetic and Environmental Factors

**Figure 1:**
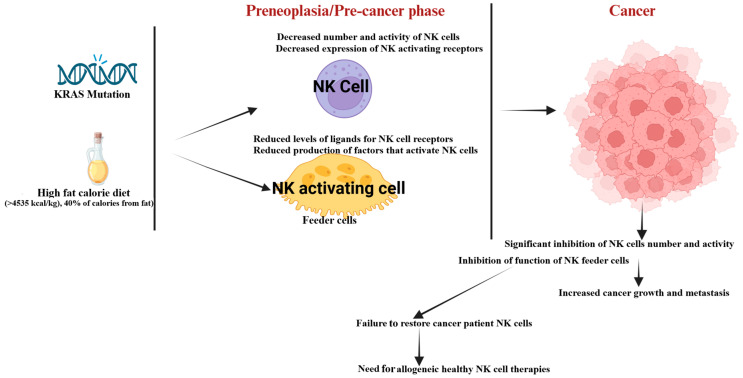
The illustration highlights how a high-fat, high-calorie diet and gene mutations impact the number and anti-cancer activity of NK cells and NK feeder cells. A decrease in the function of these cells can contribute to the onset or progression of cancer. Cancer itself further reduces the number and activity of NK cells and NK feeder cells, leading to accelerated tumor growth and metastasis. The dysfunction of autologous NK cells emphasizes the necessity of using allogeneic healthy NK cells in cancer treatment. Created with https://BioRender.com on 7 August 2025.

Genetic mutations or specific gene knockouts can result in preneoplasia or cancer [41]. Studies reveal that a high-fat, high-calorie diet (HFCD) given to healthy mice and mice with pancreatic KRAS mutations significantly reduces NK cell numbers and functions during the preneoplastic stage of pancreatic cancer [42,43]. Cytotoxic activity and cytokine secretion were impaired in KRAS-mutant mice on a control diet (CD), HFCD-fed mice, and healthy mice on HFCD [43]. Furthermore, feeder cells from KRAS-mutated mice on HFCD showed notably lower levels of MHC class I inhibitory ligands and RAE1-delta-activating ligands, essential for NK cell signaling [43]. This reduction was more significant in KRAS-mutated mice on HFCD compared to those on a CD, with WT mice on a CD showing the highest ligand levels [43]. Pancreatic intraepithelial neoplasia (PanIN) refers to a small, non-invasive precancerous lesion found in the small ducts of the pancreas. These lesions involve abnormal epithelial cell changes and are considered the main precursors to pancreatic ductal adenocarcinoma (PDAC), a highly aggressive and deadly type of pancreatic cancer. The decreased presence of MHC class I and RAE1-delta on feeder cells correlates with PanINs in KRAS-mutated mice, suggesting a link between reduced surface receptors on feeder cells, impaired NK cell activity, and PanIN development [42,43]. These findings highlight that impaired NK cell function, influenced by genetic and environmental factors during the pre-malignant phase, may play a crucial role in the onset and progression of pancreatic cancer (Figure 1).

When PDAC interact with NK cells, IL-6 secretion increases, while IFN-γ secretion decreases, potentially encouraging pancreatic tumor growth [43]. IL-6 plays a critical role in promoting PDAC proliferation and suppressing NK cell function. Studies show that adding IL-6 to tumor/NK cell cultures lowers NK-mediated IFN-γ secretion [44,45,46,47,48,49]. This indicates that adipose tissue near the tumor may shift NK cells from tumor-suppressing to tumor-promoting [8,50,51,52,53]. Blocking IL-6 could not only slow tumor progression but also reactivate NK cells suppressed by peri-pancreatic adipose tissue or tumor-infiltrating cells, offering a promising therapeutic approach for the treatment of pancreatic tumors.

The link between mutations, obesity, unhealthy lifestyles, and reduced NK function in the development of precancerous lesions in pancreatic cancer emphasizes the need for new immunotherapy strategies [42,43] (Figure 1). Understanding how lifestyle factors such as diet impact NK function underscores the importance of raising public awareness and encouraging lifestyle changes to lower cancer risks. With limited progress in enhancing NK cell function for solid tumors, innovative methods are crucial for suppressing tumor growth and for restoring NK activity. The upcoming sections will explore studies on boosting NK functions and complementary therapeutic strategies.

## 3. Reduced NK Cell Activity in the Cancer-Bearing Mice Model

Many studies show that natural killer (NK) cell cytotoxicity decreases in cancer-bearing mice. For example, mice with Lewis lung carcinoma (LLC) tumors experience suppressed NK activity within a week of tumor growth, which persists as the tumor progresses [54]. Humanized tumor-bearing mice also exhibit impaired NK cell function [42,55,56]. Similar NK cell numbers and functionality in cancer patients and hu-BLT (humanized bone marrow–liver–thymus mice) underscore the usefulness of humanized mice for studying NK–tumor interactions [56,57]. Research indicates that NK cells from oral, pancreatic, melanoma, and uterine tumor-bearing humanized mice lose significant function and exhibit reduced expansion compared to those from non-tumor-bearing mice [55,58,59]. Injecting feeder cell-activated NK cells intravenously successfully prevents tumor formation in humanized mice. Tumors in NK cell-based therapy-treated mice grow slowly, showing a differentiated phenotype, whereas those in non-treated mice grow rapidly and display a stem-like phenotype. Interestingly, tumors in NK cell-based therapy-treated mice recruit 18–22 times more human CD45+ immune cells than untreated ones [56]. Additionally, injecting NK cells restores NK cytotoxicity and enhances IFN-γ secretion in various immune cell populations, including PBMCs, splenocytes, bone marrow-derived cells, enriched NK cells, and purified T cells, in tumor-bearing humanized mice [56,58,59].

## 4. Impaired NK Cell Function in Human Cancer Patients

**Figure 2:**
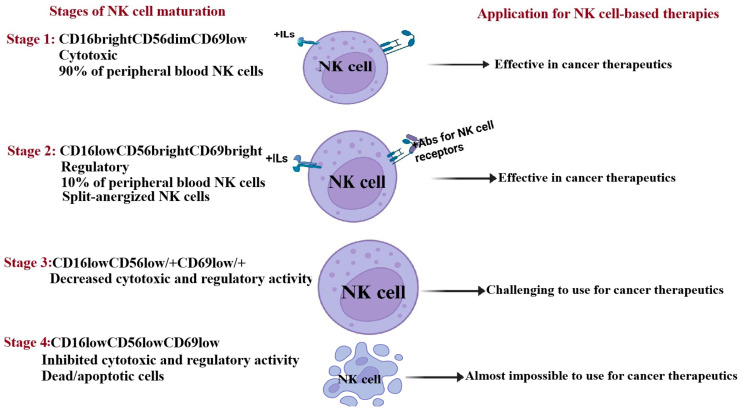
The illustration depicts the characterization of NK cells across four maturation stages, along with their potential applications in cancer treatments at each stage. Created with https://BioRender.com on 7 August 2025.

Studies have found that NK cells from peripheral blood and tumor-infiltrating sites in cancer patients often show reduced numbers, diminished anti-cancer activity, and lower cytokine secretion levels [55,60,61,62,63,64,65,66,67]. While it is well established that NK cell function is suppressed in cancer patients, the connection between this suppression and cancer development remains unclear. This raises the question of whether NK cell inhibition is a result of cancer progression or if it occurs beforehand, potentially contributing to tumor formation (Figure 1 and Figure 2).

In cancer patients, NK cells exhibit lower expression of receptors such as CD16 and NKG2D, alongside reduced survival, proliferation, cytotoxicity, and cytokine production, particularly IFN-γ secretion [55,68,69,70]. CD16 downregulation weakens antibody-dependent cellular cytotoxicity (ADCC) and recovers slowly after shedding, causing prolonged impairment [71,72]. NKG2D, which binds stress-induced ligands such as MICA, MICB, and ULBPs, undergoes clathrin-mediated endocytosis and lysosomal degradation, reducing surface expression [73,74]. Persistent stimulation of NKG2D weakens signaling, contributing to NK cell exhaustion in cancer [74]. Lower CD16 and NKG2D levels correlate with reduced IFN-γ production, which is critical for tumor suppression. CD16 shedding limits IFN-γ secretion, while NKG2D downregulation further reduces cytokine production [74,75,76]. In cancers, diminished IFN-γ levels link to NK cell exhaustion and poor outcomes [77,78]. The co-stimulation of CD16 and NKG2D enhances sustained Ca^2+^ signaling, stabilizes immunological synapses, and boosts cytotoxicity [79]. Although repeated CD16 activation reduces perforin secretion, subsequent NKG2D stimulation restores degranulation, showing adaptability [80]. These impairments significantly weaken NK cells’ ability to combat aggressive tumors, allowing tumor proliferation [81,82,83,84,85]. Suppression or death of NK cells due to the tumor microenvironment has been observed, along with reduced function during co-culture with tumors in vitro [86,87,88]. Immunosuppressive molecules such as IDO, PGE2, TGF-β, prostaglandins, and IL-10 from the tumor microenvironment inhibit NK cell activity and downregulate activating receptors [81,82,83,84,85]. Additionally, diminished function and expression of NK cell receptor ligands on feeder cells in cancer patients highlight the need to address these factors for improved cancer treatment outcomes [58] (Figure 1).

The reduced function of peripheral blood-derived NK cells and the limited presence of tumor-infiltrating NK cells are associated with poor outcomes in cancer patients [55,62,63,89,90,91,92]. To address this, various in vitro techniques have been developed to increase the number and activity of NK cells, allowing for higher therapeutic doses in cancer treatment [93,94,95,96,97]. Engineered molecules combining NKG2D extracellular domains with anti-CD16 Fab fragments boost NK cell activation, degranulation, and IFN-γ production against AML cells, offering promising therapeutic approaches [98,99]. Restoring or preventing the loss of NK cell activating receptors can help maintain the balance between activation and inhibition [100,101,102].

## 5. The Maturation Stages of Natural Killer Cells

Activating NK cells is a complex process that involves multiple maturation stages in humans, characterized by specific surface markers that define their subsets, activation, and tissue residency [50,103]. Key markers such as CD16, CD56, and CD69 distinguish NK cell populations with unique functions and characteristics [104,105,106,107,108]. These cells are divided into two subsets based on CD56 and CD16 expression [105]. CD56^dim^CD16^+^ NK cells, the majority in peripheral blood (90–95%), are highly cytotoxic, equipped with perforin and granzyme B, mediating ADCC and targeting virus-infected or tumor cells. CD56^bright^CD16^−^ NK cells, found in secondary lymphoid tissues and some peripheral tissues, have low cytotoxicity but are proficient in producing cytokines such as IFN-γ, TNF-α, TNF-β, GM-CSF, IL-10, and IL-13 [6,104,109]. They play immunoregulatory roles, interacting with dendritic cells and T cells, expressing inhibitory receptors such as NKG2A, low KIR levels, and lacking mature cytotoxic granules [6,104]. TNF-α and IFN-γ production by NK cells significantly influences tumor differentiation, leading to slower growth and metastasis in differentiated tumors compared to stem-like tumors [56,58,110]. CD69, alongside CD49a, CXCR6, and CD103, marks early activation and tissue residency, appearing on NK cells in organs such as the liver, lungs, skin, gut, and uterus [106,111,112,113]. CD69 is typically expressed on CD56^bright^CD16^low/−^ NK cells, which are less cytotoxic but secrete regulatory cytokines for immune modulation and tissue repair. Activated peripheral blood NK cells can also express CD69. Overall, CD56^dim^CD16^+^ NK cells are primary cytotoxic effectors in circulation, while CD56^bright^CD16^−^NK cells regulate immune responses, and CD69+ tissue-resident NK cells focus on local immune surveillance and modulation [105,114].

To understand the diverse roles of NK cells in contexts such as the tumor microenvironment or during infections, it is crucial to explore their regulation and mechanisms. Studies have identified four stages of NK cell maturation in humans based on CD16, CD56, and CD69 surface receptors [67,115] (Figure 2). Stage 1 features CD16^bright^CD56^dim^CD69^low^ NK cells, which make up about 90% of peripheral blood NK cells and are crucial for targeting and destroying stem-like cancers. Stage 2 includes CD16^low^CD56^bright^CD69^bright^ NK cells, which are less cytotoxic or anergized, aiding in regulating other cell functions and promoting tumor cell differentiation through secreted factors. In Stage 3, NK cells may lose cytotoxicity or the ability to produce IFN-γ as they mature further. Stage 4 marks the apoptosis of NK cells (Figure 2).

Among the four stages, stages 1 and 2 are crucial in preventing tumor growth and spread by directly killing and promoting tumor differentiation through secreted cytokines, respectively. At stage 2, NK cells are termed “split-anergized NK cells” because they reduce their cytotoxic functions while releasing higher levels of cytokines such as IFN-γ and TNF-α [89,116,117]. This split anergy can be induced in NK cells through IL-2 treatment and anti-CD16 monoclonal antibodies, mimicking activation during interactions with tumor cells [103,116,117]. The tumor microenvironment (TME), including monocytes, Myeloid-Derived Suppressor Cells (MDSCs), and immunosuppressive cytokines such as TGF-β, IL-10, and PGE2, helps induce and maintain split anergy. Unlike T-cell anergy, which suppresses all functions, NK-cell split anergy specifically reduces cytotoxicity while increasing cytokine and chemokine secretion [89,118,119]. The enhanced secretion of immunoregulatory and inflammatory cytokines is vital for the differentiation of stem-like tumors [120,121]. The downregulation or loss of CD16 receptors and reduced activity of cytotoxic granule components such as granzyme B and cathepsins impair NK cells’ ability to kill stem-like cancers. This loss of cytotoxicity is key to tumor differentiation and may lead to NK cell deactivation when encountering well-differentiated tumor cells [89,120,122]. Split-anergized NK cells slow cancer growth and metastasis, as differentiated tumors grow and spread more slowly than stem-like tumors. They also improve the effectiveness of other therapies, as differentiated tumors are more sensitive to chemotherapy, checkpoint inhibitors, radiotherapy, and CD8+ T-cell-based treatments [7,53].

There is a noticeable increase in stage 3 NK cells during preneoplasia or in the contexts of high-fat diets and obesity. In cancer patients, NK cells display a stage 4 phenotype, which compromises their function [123] (Figure 2). Studies reveal reduced NK cell cytotoxicity in both the tumor microenvironment and peripheral blood, along with decreased CD16 receptor levels on NK cell surfaces. NFκB, STAT3, and COX2 signaling in tumor and immune cells create an immunosuppressive environment that fosters NK dysfunction. Tumor cells produce ligands and secrete factors that downregulate activating receptors while upregulating inhibitory signals on NK cells. NK cells exposed to the TME exhibit impaired expression of cytotoxic receptors (NKG2D, NKp30, NKp46) and diminished cytolytic granule activity. This weakened NK cell function is linked to higher cancer risk, while improved NK cell activity and greater tumor infiltration correlate with better outcomes.

## 6. Memory-like NK Cells

Over the past decade, research has revealed that NK cells can develop memory or memory-like properties, allowing them to recall previous encounters and respond more effectively to pathogens or stimuli [124,125,126,127]. Memory NK cells are a unique subset of innate lymphocytes with adaptive-like, long-lasting responses to specific infections or triggers, which have significant potential for immune defense and cancer therapy [125,128]. These cells come in two main forms: memory NK cells, which show antigen specificity, longevity, and strong recall responses such as adaptive immunity; and memory-like NK cells, which have enhanced responses but may lack clear antigen specificity or long-term persistence [128,129,130,131]. Memory NK cells can expand clonally and form long-lived populations after infections such as cytomegalovirus (CMV) [132,133]. For instance, in mice infected with murine CMV (MCMV), NK cells with the receptor Ly49H expand greatly before contracting into a lasting memory pool that responds strongly to secondary infections [134,135,136]. In humans, NKG2C+ NK cells expand after human CMV (HCMV) infection and undergo epigenetic changes resembling memory features [137,138,139,140]. Additionally, cytokine-induced memory-like (CIML) NK cells can be generated through brief activation with cytokines such as IL-12, IL-15, and IL-18, reprogramming them to last longer and produce higher levels of IFN-γ upon restimulation, even without specific antigen exposure [141,142,143,144]. This phenomenon, observed in both mice and humans, shows great promise for immunotherapy. Memory and memory-like NK cells offer key advantages over conventional NK cells, including enhanced cytotoxicity and cytokine production (especially IFN-γ), epigenetic reprogramming with DNA methylation changes in IFN-γ gene regulatory regions supporting durable functional changes, and improved proliferation and persistence in vivo [128,145,146].

Memory-like NK cells are showing promise in clinical trials, particularly in cancer immunotherapy [128]. Cytokine-induced memory-like NK cell therapies have shown safety, in vivo persistence, and temporary disease control in both hematologic cancers and solid tumors such as head and neck cancer [141,142,147,148,149]. Their enhanced functionality makes them attractive for adoptive cell therapies, addressing challenges such as poor persistence and exhaustion, as seen in traditional NK cell treatments. Understanding NK cell memory mechanisms helps develop strategies to boost antitumor immunity through cytokine combinations, checkpoint inhibitors, and genetic engineering. Memory NK cells blur the line between innate and adaptive immunity by offering both antigen-specific and antigen-independent responses after activation [129,150]. They provide strong, long-lasting protection against infections such as CMV and open up exciting new possibilities for cancer immunotherapy. With ongoing research and early trials, their unique abilities are gaining attention for improving immune-based treatments. Memory and memory-like NK cells mark a major shift in immunology and immunotherapy, combining the speed of innate responses with the precision and durability of adaptive immunity [129].

## 7. Progress and Challenges in Developing NK Cell-Based Cancer Immunotherapies

Recent clinical trials have shown the improved effectiveness of cancer immunotherapies for various types of cancer [39,151,152,153,154]. NK cell therapies are generally well tolerated, with low risks of cytokine release syndrome or GVHD, making them a safer alternative to T-cell-based therapies [155,156]. Clinical outcomes depend on factors such as cancer type, NK cell source (autologous or allogeneic), expansion methods, dosing, and combination treatments [155,156]. Long-term success relies on improving persistence, trafficking, and overcoming suppression from the tumor microenvironment (TME). NK therapies show promise in treating blood cancers such as AML, multiple myeloma, and lymphoma, with adoptive transfer leading to significant responses and lower relapse rates post-transplant [156]. However, their effectiveness in solid tumors remains inconsistent due to poor tumor infiltration, limited persistence, and TME-induced dysfunction [157]. Trials involving over 600 solid tumor patients report overall response rates of about 28% and disease control rates around 63%, indicating moderate antitumor activity with variability [158]. Since their discovery, researchers have worked on safe and effective ways to use NK cells in treating cancer. These cells can be sourced from blood, cord blood, stem cells, or induced pluripotent stem cells, then expanded and cryopreserved for ready-to-use applications [28,159,160]. This section explores advancements in activating NK cells through gene manipulation, creating NK-cell-based immunotherapies using feeder cells, cytokines, and genetic modifications, along with the challenges faced.

### 7.1. Modifying the Genes of NK Cells to Enhance Their Activity

NK-cell knockout (KO) and knock-in (KI) techniques are designed to address functional limitations, improve expansion and persistence, and enhance antitumor activity in cancer immunotherapy [161]. CRISPR-Cas9 technology, particularly with electroporation of Cas9 ribonucleoproteins (RNPs), allows precise genome editing in both primary and expanded NK cells, though challenges persist [162,163]. Research has explored the impact of tumor gene knockouts on NK cell function [164]. For example, knocking out NKG2A (encoded by KLRC1), an inhibitory receptor for HLA-E on tumors, results in only slight cytotoxicity improvements that are often inconsistent and statistically insignificant [165,165,166]. Similarly, CD96 knockout has mixed effects on cytotoxicity, while knocking out Casitas B cell lymphoma-b (an E3 ubiquitin ligase) provides modest benefits [167,168]. On the other hand, knocking out genes such as TGF-β strengthens NK cell function by countering its immunosuppressive effects, and knockout of suppressor of cytokine signaling 3 enhances NK cell expansion and cytotoxicity [161,169,170]. CD38 knockout prevents NK cell fratricide caused by anti-CD38 antibodies, potentially improving in vivo persistence [171,172]. Chimeric antigen receptor (CAR) constructs enable MHC-independent targeting through extracellular scFv to recognize tumor antigens, linked to intracellular activation domains such as CD3ζ, 4-1BB, and CD28. CAR-NK cells offer improved tumor specificity and cytotoxicity, with lower risks of graft-versus-host disease or cytokine release syndrome compared to CAR-T cells [173]. Lentiviral transduction or electroporation of CAR mRNA are commonly used to engineer NK cells with CARs [174,175].

Glioma cells release miR-1983 enclosed in exosomes, shielding it from degradation and enabling it to serve as a messenger between cells, influencing immune responses in the tumor microenvironment [176,177,178]. miR-1983 is primarily present in myeloid cells, such as plasmacytoid and conventional dendritic cells, acting as a TLR7 ligand to activate NK cells by binding to TLR7. This interaction triggers signaling through the MyD88-IRF5/IRF7 pathway, leading to interferon-beta (IFN-β) production [179]. IFN-β binds to the IFNAR1 receptor on NK cells, activating them to attack and destroy glioma cells [180]. This creates an innate miR-1983-TLR7-IFN-β-NK cell antitumor circuit, which operates before adaptive immunity is engaged [179]. IFN-β signaling through IFNAR1 prepares NK cells for cytotoxic activity, mainly through perforin-dependent mechanisms, with IFN-γ playing a smaller supporting role. Knockout mice missing TLR7, MyD88, IRF5, IFN-β, or IFNAR1 show reduced tumor rejection, highlighting the pathway’s significance [179,181]. Enhancing the miR-1983-TLR7-IFN-β pathway could boost NK-mediated glioma elimination and offer promise for other cancer treatments. Overcoming tumor immune evasion involves blocking inhibitory receptors (e.g., TIGIT, PD-1, and NKG2A) and targeting suppressive tumor metabolites to enhance NK activation [182]. Using specialized NK cell subsets, such as g-NK cells (FcεRIγ-negative) with strong ADCC capacity, combined with monoclonal antibodies, offers synergistic tumor killing [183].

### 7.2. Feeder Cells Triggered the Activation and Expansion of NK Cells

Various methods have been developed to tackle the challenge of expanding NK cells ex vivo, often involving feeder cells with or without cytokines and other activation signals [55,93,94,95,96,184,185,186,187,188]. Feeder cells play a key role in activating and proliferating NK cells by providing receptor–ligand interactions and cytokine support, enabling large-scale therapeutic use [189,190,191]. The K562 cell line, derived from human erythroleukemia, is a common feeder system for NK cell expansion [189]. Autologous or allogeneic PBMCs, depleted of CD3+ cells and sometimes enriched with CD4+ T cells, can also act as feeder pools, releasing stimulatory factors [192]. Studies have shown osteoclasts as effective feeder cells for NK cell expansion [55]. Myeloid subsets are essential in activating NK cells, enhancing their cytotoxicity and cytokine secretion compared to monocytes and dendritic cells [193]. These advancements in expansion techniques have unlocked new therapeutic opportunities [95,194]. NK cell-based therapies are further boosted by combining them with checkpoint inhibitors such as anti-PD1 monoclonal antibodies for cancer treatment [195,196]. The effectiveness of these therapies has been demonstrated in vivo through humanized mouse models, showing potential for clinical applications [42,55,56,57,58,63,91,197,198,199,200,201,202,203,204,205,206,207,208,209,210,211]. Additionally, technologies include γ-irradiating feeder cells before co-culture to prevent their proliferation while maintaining their stimulatory function [212]. Feeder cell cultures are often supplemented with cytokines such as IL-2, IL-15, and IL-21, along with membrane-bound cytokines [213,214].

### 7.3. Genetically Engineered Feeder Cells to Stimulate NK Cell Activation and Expansion

Genetic engineering of feeder cells to express 4-1BBL and IL-15 or IL-21 has become a popular method for NK cell expansion [55,95,215,216,217,218,219,220,221,222]. The K562 cell line is frequently modified to express stimulatory molecules and cytokines such as mbIL21, mbIL15, and 4-1BBL (CD137L) or combinations with CD80, IL-2, or IL-12p35 [189]. These engineered feeders provide strong activation signals through receptor–ligand interactions (e.g., 4-1BB on NK cells) and robust proliferation via cytokine signaling. K562.mbIL21.4-1BBL achieves ~48,000-fold expansion in 21 days while maintaining telomere length, supporting long-term proliferation for clinical manufacturing [189]. K562.mbIL15.4-1BBL supports ~800-fold expansion but exhibits telomere shortening, limiting growth beyond 4–6 weeks [189,223]. Variants such as K562 cells expressing mbIL2 or mbIL13 enhance activation and cytotoxicity [224]. Feeders co-expressing mbIL21, mbIL15, and 4-1BBL enable rapid, large-scale clinical-grade NK expansion (~17,900-fold in 2 weeks) with improved tumor-killing abilities in vitro and in vivo [222]. Some feeders incorporate IL-12p35 to further boost NK expansion and cytotoxicity [225]. Specific gene knockdowns, such as NFkB in HEp2 tumors, CD44 in breast and melanoma tumors, or COX2 in various cells, significantly enhance NK cell functionality [51,53,164,226]. Knockout mice affecting inflammation and NK signaling show heightened responsiveness [227]. EBV-Transformed Lymphoblastoid Cell Lines (EBV-LCLs), created through Epstein–Barr virus transformation, naturally express ligands such as 4-1BBL, CD155, CD48, and CD58, which engage with NK-activating receptors [228,229]. These cells can achieve 1000–10,000-fold expansions within 2–3 weeks and are commonly used in clinical-grade NK cell production to sustain activation and functional characteristics [228,229]. They are often paired with cytokines such as IL-2 and IL-21 to boost expansion [230]. Additionally, genetically engineered T cell lines, such as transformed HuT 78 cells expressing 4-1BBL, IL-21, and TNF-α, have been developed as feeder cells, enabling significant NK expansion and strong antitumor effects [192]. Repeated stimulation with these feeders can lead to over 700-fold expansions in just a few weeks [192].

### 7.4. Cytokine-Driven NK Cell Expansion

Cytokine-induced NK cell expansion plays a key role in adoptive cell therapy for cancer treatment [231,232]. IL-2 supports NK cell survival, activation, and proliferation, while IL-15 is essential for NK development and survival, boosting cytotoxicity and expansion, often through feeder cell-bound delivery [213]. IL-21 enhances NK maturation, expansion, and cytotoxicity, particularly when delivered via feeder cells [233]. IL-12 and IL-18 promote cytokine production and generate memory-like NK cells with better persistence and antitumor activity [234]. Although cytokine cocktails without feeder cells can expand NK cells, they typically result in less than 100-fold expansion over several weeks [235,236]. Combining cytokines with feeder cells greatly improves NK expansion and functionality. The IL-12, IL-15, and IL-18 cocktail is used for short-term activation (12–18 h) to produce cytokine-induced memory-like (CIML) NK cells, which show increased IFN-γ production, proliferation, persistence, and tumor cytotoxicity [143]. CIML NK cells hold promise for improved in vivo persistence and function [141]. Additional cytokines, such as IL-3, Flt3 ligand (FL), Kit ligand (KL), IL-7, and IL-10 influence NK development and function depending on the environment and developmental stage [237]. Ex vivo expansion protocols use cytokine cocktails, often with feeder cells and culture systems, to produce clinically viable NK cell doses [238]. Early clinical trials demonstrate safety, strong in vivo expansion, and potential effectiveness of cytokine-driven NK cell therapies for hematologic cancers [239].

### 7.5. Challenges in Developing and Ensuring the Efficacy of NK Cell-Based Therapies

NK-cell-based therapies are known for their safety, but their effectiveness remains a challenge due to various issues. While T cells comprise 40–60% of lymphocytes in human peripheral blood, NK cells only account for 5–15% [240]. As outlined in Section 2, Section 3, Section 4 and Section 5, NK cell functions are influenced by many factors, especially in preneoplasia and cancer [42,204]. Autologous NK cells from cancer patients are often impaired due to prior immunosuppression, and their expansion presents unique challenges compared to healthy donors (Figure 1 and Figure 2). Rapid T cell proliferation in cancer patients interferes with NK cell expansion, lowering the cytotoxic function of the expanded cells [55]. This highlights the critical interaction between NK cells and T cells, especially Tregs and MDSCs, which hinder NK cell function [241]. Comparative studies show that NK cells expanded from cancer patients using the same methodology as healthy donors exhibit reduced cytotoxicity and IFN-γ secretion levels [55,63]. Patient-derived NK cells also display decreased expression of activating receptors such as CD16, CD56, Nkp30, Nkp44, Nkp46, NKG2D, and CD54 [55]. Expanding large, potent NK cell populations ex vivo is difficult, limiting the scalability of these therapies. Cryopreservation and thawing processes reduce their viability and functionality after infusion.

Gene knockdown has been proven to enhance NK cell activation, whether applied to the target cells or the NK cells themselves [53,164]. However, many NK cell expansion methods produce low-quality cells, failing to achieve both robust growth and retention of functionality [240]. Adoptively transferred NK cells often struggle with survival and proliferation in vivo. Expanding transferred NK cells in vivo is difficult, especially in the immunosuppressive TME. Tumors can downregulate activating ligands or release soluble ligands (e.g., MICA/B), engaging inhibitory NK receptors and causing dysfunction [157,242]. Inhibitory checkpoint molecules such as NKG2A, TIGIT, and PD-1 also lead to exhaustion and reduced cytotoxicity [243]. Physical barriers and low chemokine expression further hinder NK cell recruitment and infiltration into solid tumors [157]. NK cells lack tumor specificity, relying on germline-encoded receptors, making them less precise than T cells [86]. Tumors may evade NK-mediated killing by maintaining or increasing MHC class I molecules, activating inhibitory KIRs on NK cells [244]. Gene editing and CAR engineering in NK cells are hindered by issues such as low transduction rates and inconsistent editing efficiency [245]. Challenges remain in achieving efficient gene transduction, stable expression, and identifying optimal tumor-specific antigens, even with modifications such as CAR-NK [246]. Feeder layer-dependent approaches, including K562, have shown limited success in sustaining NK cell activation over time [55,197]. Cytokine therapies, including IL-2 and IL-15, can activate NK cells but often lead to severe toxicities such as vascular leak syndrome and cytokine release syndrome [247]. While cytokines show potential for significant NK cell expansion, variability in donor-derived NK cell performance remains a key limitation, further complicated by the search for super donors [231]. Though NK cells pose a lower GVHD risk compared to T cells, unpredictable immune responses still present challenges [248]. Their heterogeneity, with phenotypes such as CD56^bright^ vs. CD56^dim^ and tissue-resident vs. circulating, complicates standardization and therapeutic predictability [249]. Furthermore, NK cells are rapidly inactivated in the tumor microenvironment, even after cytokine-induced activation [250].

The limited in vivo persistence of NK cells after infusion is a major challenge, especially against solid tumors, as they often face depletion or exhaustion caused by the suppressive TME and host immune rejection [157]. Factors such as TGF-β, IL-10, hypoxia, elevated adenosine, reactive oxygen species, prostaglandins, and suppressive cells (Tregs, MDSCs, TAMs) reduce NK cell survival and activation [251,252,253]. Addressing these challenges is crucial for enhancing NK cell longevity. Combining NK therapies with immune checkpoint inhibitors, radiotherapy, DNA-damaging agents, or oncolytic viruses shows early potential in overcoming these barriers and improving outcomes [254].

Allogeneic NK cells, while advantageous for adoptive therapies due to their lack of GVHD risk, often face challenges such as immune rejection or limited persistence caused by HLA mismatches [39,40]. A major challenge of allogeneic NK cell therapy is that most recipients have functional immune systems capable of recognizing and rejecting these foreign cells [29]. Host T cells may attack donor cells with mismatched HLA molecules, B cells can produce alloantibodies tagging NK cells for destruction, and host NK cells may target donor cells lacking self-HLA ligands [255,256]. Additionally, macrophages and complement activation contribute to eliminating donor NK cells [257]. This rejection limits the persistence and effectiveness of NK cell therapies, especially as the host immune system recovers post-lymphodepletion [258]. To combat this, pre-conditioning with lymphodepletion (e.g., cyclophosphamide, fludarabine) can temporarily suppress the host immune response, though it has short-term effects and toxicity [258,259]. Other approaches include genetically engineering donor NK cells to reduce HLA expression (e.g., knocking out beta-2 microglobulin to remove HLA-I) and adding non-polymorphic HLA molecules (HLA-E, HLA-G) or “don’t eat me” signals (CD47) to evade host attacks [260,261]. Advanced gene editing tools such as multiplexed CRISPR/Cas9 create “immune-evasive” NK cells with extended durability [169]. Choosing donors based on KIR and HLA compatibility can boost NK cell function and minimize rejection, while repeat dosing of off-the-shelf NK products could enhance therapeutic outcomes [262].

To address these challenges, future strategies aim to develop memory-like NK cells, such as cytokine-induced memory-like NK cells, to enhance their persistence and antitumor activity. Efforts involve engineering NK cells with enhanced CAR constructs that incorporate NK-specific signaling domains and cytokine-support genes such as IL-15/IL-15Rα [263]. Other methods involve combining therapies with immune checkpoint inhibitors, TME-modulating agents, or NK cell engagers to boost infiltration and functionality. Additionally, refining manufacturing protocols can improve the recovery and potency of cryopreserved products after thawing.

## 8. Conclusions

Recent advancements in cancer research have highlighted the vital role of NK cells in fighting cancer. Studies show a significant decline in NK cell function in preneoplastic individuals and cancer patients, stressing the need to restore NK cell activity for effective treatment. The immune landscape in cancer patients, especially significant dysfunction of NK cells, highlights the significance of choosing allogeneic NK cell immunotherapy. Allogeneic NK cell-based therapies hold promise for effectively eradicating tumors. However, these therapies face challenges such as short persistence and expansion after infusion, poor tumor infiltration, suppression by the tumor microenvironment, limited tumor specificity, manufacturing hurdles, and treatment-related toxicities. Tackling these issues calls for advanced genetic engineering, improved ex vivo and in vivo stimulation techniques, and complementary immunomodulatory therapies.

## Data Availability

No data was generated for this article.
